# Annotations

**Published:** 1891-04-25

**Authors:** 


### Annotations.

**Logical Women.**

What a blessing it is that women are developing a little taste for the study of logic! Such a taste portends a revolution in the household; for women are no mere word-spinners, and "learning for learning's sake" vapidities. What they know they cannot help putting into practice under the pressure of the daily stress and strain of household business. It seems, from a correspondence now being carried on in the pages of a contemporary, that "grave doubt is cast upon the fitness of logical women for domestic life." This will make experienced husbands rub their eyes, or their spectacles if they wear any. Logical women not fit for domestic life! Are illogical women then fit for domestic life? Is it not the case that ninety-nine of every hundred domestic troubles spring from this one source alone—the want of logic in the wife? If a woman fails to make her weekly allowance suffice, it is because she is not logical; if she spends three guineas on a bonnet when ten or fifteen shillings would be enough it is because she is not logical; if she has cold meat for dinner oftener than once a week it is clearly because she is not logical. "Of course," says a lady logician, "there always will be a class of men who do not desire to have their actions and habits too closely scrutinised, or their transparent excuses detected by the clear eyes of a thoughtful logical partner." Well, yes! There probably will always be a considerable number of this unlucky class, though it may be hoped that as "thoughtful logical partners" increase in number the shuffling husbands who find their "transparent excuses detected," will also find it expedient to betake themselves to some quiet nook in a neighbouring forest, where a little prussic acid may be invoked to give them "surcease of strife." "A straightforward, honourable husband," continues our lady logician, "never yet found his comfort impaired by his wife's ability to conduct an argument either with himself or a refractory Jehu." We do not feel quite so sure of that; the statement needs prolonged consideration, and possibly a good deal of qualification. About the "refractory Jehu" there need be little doubt. The man who has a wife that can promptly settle a cabman is a man to be envied; but when it comes to settling one's self, with logic of even the most Kantian character, one has a most illogical desire to demur. Of course this is unreasonable. A Roman nose is a Roman nose, and a logical wife is a logical wife, and he who has either the one or the other must try to live up to his privileges. But we men ought not to be taken too hurriedly along these new lines of development. We are but "poor human nature" after all, and we must be borne with. We most fully appreciate the logic, especially when it is applied in the kitchen, and to cabmen at the door. But we ourselves will, if we may, choose the results rather than the processes. A good downright "yes" or "no" of the old-fashioned sort, will convince us quite as readily as the use of a feminine "sorites." Brains let us have in our wives, and reason, and common sense; but not too much, at any rate as yet, of Whately or Mr. John Locke.

**The Radcliffe Infirmary, Oxford.**

Last week we published an energetically expressed protest from the medical staff of the Radcliffe Infirmary, Oxford. We published also a leader which had been passed for the press before the protest of the Radcliffe staff reached us. It is possible that, had the protest arrived sooner, some modification of the leader might have been made. That would have been a pity; because it is well that those who are interested in the affairs of the Radcliffe Infirmary should know how the report of our special Commissioner affected a medical writer who has never seen the Radcliffe Infirmary at all, and who wrote exactly what he felt after reading our Commissioner's report. Regular readers of The Hospital are well aware that though we sometimes speak strongly we have no penchant for either spleen or malice. The conductors of a newspaper owe it to themselves and to the public to be both honest and courageous. There is no doubt that the Radcliffe Infirmary, though it is not falling into ruins, nor smothered in dirt, nor entirely destitute of some modern sanitary appliances, yet falls a good deal short of a just standard of modest external beauty, internal fitness, and sanitary excellence. Now that Infirmary, being at Oxford, and being connected with our greatest University, ought not to be less than excellent in any one department. What our Commissioner affirmed was, that it is less than excellent in several departments, and those very important ones; and the letter of the staff, which admitted the facts stated about the external appearance of the hospital, the entrance in the basement, the character of three wards containing together forty-five beds—that is nearly half the beds of the hospital—and the discreditable Parian cement, constitutes, it seems to us, quite a sufficient confirmation of our Commissioner's views. The truth is that Oxford is bound by every consideration of science, culture, and religion, to have a model hospital for the sick. We are surprised exceedingly that the medical staff should have taken exception to our Commissioner's criticism. On the sanitary ground alone they should have ranged themselves on our side of the question. No doctor's mind should admit such a principle as "relative sanitary excellence" for a hospital. The only standard for all hospitals is that of "absolute sanitary perfection." A hospital that is not sanitarily perfect, is insanitary and injurious. A great University like Oxford is nothing if not a standard-bearer. Absolute excellence and absolute perfection should at least be its ideals; and where those ideals can be realised, as they can in hospital construction, they must be realised. It is plain that Oxford owes it to itself to build anew the older portions of the hospital, with a new medical school, which shall be of such a character as that the whole country can look to it as a model and an example. Until this is done the city of colleges and churches, of learning and of wealth, must feel that, in one respect at least, it does not represent either the highest culture or the noblest religion of the times. We are glad to see that Dr. Bright, the Treasurer of the Hospital, and Master of University College, makes in the *Oxford Chronicle*, the practical comment upon our Commissioner's report, of asking for £10,000, to spend in making the improvements which he sees the Infirmary needs. This is sensible, and proves Dr. Bright to be a man of the world, as well as the learned head of a college. It would be better to follow the example of Derby, Edinburgh, and other places, and to pull the old hospital down, and raise a model building in its place. We hope Dr. Bright and the other managers will take this view of the situation, and give us the privilege of helping them by all the means in our power to raise the necessary funds.

**The Tomb of Aristotle.**

Medical readers ought to be interested in Dr. Waldstein's interesting discovery in Bœotia. The Cambridge archæologist has been personally conducting excavations, and he believes that he has come upon the genuine tomb of Aristotle. All students who build safely, because they build upon facts and experience, would be glad to see even the grave of so rare a leader. The medical men of this country have not made much of Aristotle, and yet it is probable that to his early medical training the world owes the practical direction which was given to science by his genius, and which, in spite of all the scientific skimmers and triflers who have played with science since his day, has never been wholly lost. Aristotle belonged to a medical family, although there is no evidence of his ever having practised the medical art as a profession. He was, however, even in his mature life, a great dissector of animals. It is possible that a good many people regard Aristotle as they do some unknown medical Smith or Brown who wrote professional treatises twenty years ago—that is, they think him "out of date." No reader who is capable of taking this view of the facts is likely to be converted by such a brief argument as could be put into one of our annotations. But Aristotle can never be less than one of the greatest writers of what might be called, if such a book existed, the "World's Scientific Bible." Of course, he did not always know facts as we know them; but the man who knows nothing but facts is always the most intolerable pedant and the greatest nuisance of every department of human activity. Aristotle possessed in an unique degree certain endowments which, thanks to their hard material education, so many medical men lack: he was a man of "light and leading." Even the many keen intellects of his own time were not able to catch his royal spirit and to chain it down within the prison of their own "wordspinning." The most honourable thing that could be said of the medical profession of to-day would be that it was the intellectual successor of Aristotle, and that it was worthy of its great leader.

